# Bronchogenic Cyst with Degeneration of the Adjacent Membranous Portion of the Trachea: A Case Report

**DOI:** 10.70352/scrj.cr.25-0287

**Published:** 2025-08-20

**Authors:** Takamitsu Hayakawa, Mikako Mitake, Hirohisa Inaba, Mayumi Kobayashi, Yasuhiro Watanabe, Asako Okabe, Kazuhito Funai

**Affiliations:** 1 Department of Thoracic Surgery, Japanese Red Cross Shizuoka Hospital, Shizuoka, Shizuoka, Japan; 2 Department of Anesthesia, Japanese Red Cross Shizuoka Hospital, Shizuoka, Shizuoka, Japan; 3 Department of Diagnostic Pathology, Japanese Red Cross Shizuoka Hospital, Shizuoka, Shizuoka, Japan; 4 First Department of Surgery, Hamamatsu University School of Medicine, Hamamatsu, Shizuoka, Japan

**Keywords:** bronchogenic cyst, tracheal stenosis, complete resection

## Abstract

**INTRODUCTION:**

Bronchogenic cysts are congenital, benign cystic lesions that develop in the mediastinum. Many patients are asymptomatic, and conservative observation is often chosen in clinical practice. However, delayed surgical resection following cyst enlargement and compression of the adjacent membranous portion of the trachea can result in perioperative challenges.

**CASE PRESENTATION:**

We report the case of a 53-year-old woman who had been under observation for 10 years for an asymptomatic mediastinal mass. The mass enlarged gradually and caused persistent cough along with obstructive ventilatory impairment. Chest CT revealed a 5.5 cm mass compressing the membranous trachea, resulting in tracheal stenosis. MRI revealed a homogeneously high T2 signal within the mass, suggesting a simple cystic nature. PET showed no accumulation of fluorodeoxyglucose in the mass, indicating no malignancy. Based on preoperative diagnosis of a bronchogenic cyst, the patient underwent video-assisted thoracoscopic surgery. Tracheal intubation using a double-lumen tube was challenging due to the tracheal stenosis. Moreover, the membranous trachea compressed by the cyst exhibited white degeneration, suggesting thinning and fragility. Intraoperatively, due to firm adhesion to the membranous trachea, a part of the cyst wall was intentionally left in place to avoid tracheal injury. The inner lining of the residual cyst was cauterized to prevent recurrence. Bronchoscopic findings on POD 7 showed that white degeneration of the membranous trachea remained. Histopathological examination revealed ciliated columnar epithelium and cartilage on the cyst wall, confirming the diagnosis of a bronchogenic cyst.

**CONCLUSIONS:**

Long-term observation of mediastinal bronchogenic cysts can lead to degeneration and thinning of the membranous trachea, increasing the risk of tracheal injury and incomplete resection during surgery. Therefore, the absence of symptoms should not justify delaying surgical intervention. Preoperative assessment for coexisting malignancy and tracheal abnormalities can support surgical decision-making to ensure a safe procedure.

## Abbreviation

FEV1.0
: forced expiratory volume in one second

## INTRODUCTION

Bronchogenic cysts are benign congenital masses that develop in the mediastinum or pulmonary parenchyma due to abnormal budding of the foregut during embryogenesis. Many patients with mediastinal bronchogenic cysts are asymptomatic^1,2)^ and, in clinical practice, are followed conservatively without surgery. We encountered a case in which long-term observation revealed white degeneration in the membranous portion of the trachea compressed by the cyst, appearing thin and fragile. Concerning tracheal injury during surgery, complete resection of the cyst could not be achieved. We present this case because few previous reports have documented alterations in adjacent organs caused by bronchogenic cysts.^1)^

## CASE PRESENTATION

**Fig. 1:**
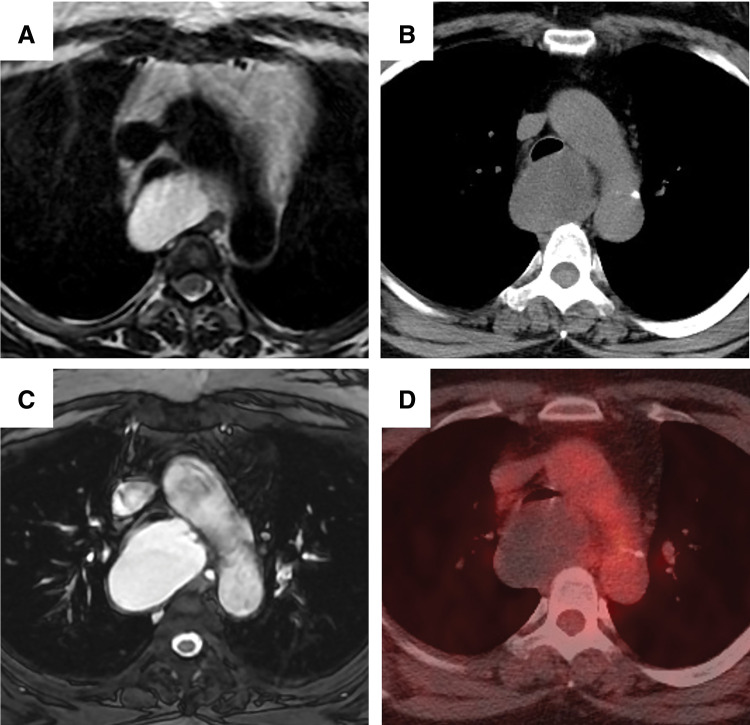
Radiological findings. (**A**) MRI performed 10 years prior to the surgery demonstrates a uniform cystic lesion. (**B**) Preoperative chest CT shows a well-defined mass in the middle mediastinum. (**C**) Preoperative T2-weighted MRI demonstrates a uniform high signal inside the mass. Compared with the MRI obtained 10 years earlier, the mass is enlarged, and the tracheal stenosis is more severe. (**D**) Preoperative PET reveals no accumulation within the mass.

A 53-year-old woman, a non-smoker, had been under observation for 10 years after a mediastinal mass was incidentally detected on CT. At that time, MRI revealed a uniform cystic lesion measuring 4.4 cm in diameter, compressing the trachea (**Fig. 1A**). Because the patient remained asymptomatic, conservative follow-up was continued. Although the mass was not infected during this period, it gradually increased in size. Eventually, the patient developed a persistent cough. Pulmonary function testing revealed the development of obstructive ventilatory impairment (forced expiratory volume in 1 s [FEV1.0]: 2.31 L, FEV1.0%:69.79%), which had not been observed before the onset of cough. Due to the emergence of symptoms and respiratory dysfunction, surgical resection was planned. Chest CT showed a well-defined 5.5 cm mass located dorsal to the trachea, compressing its membranous portion. The trachea consequently became stenotic, with the narrowest diameter measuring 5 mm (**Fig. 1B**). MRI revealed a homogeneously high T2 signal in the mass without any solid component (**Fig. 1C**). Compared with the MRI obtained 10 years earlier, the mass was enlarged, and the tracheal stenosis was more severe. PET showed no accumulation of fluorodeoxyglucose in the mass, indicating no malignancy (**Fig. 1D**). Based on these imaging findings and the anatomical location, we diagnosed a bronchogenic cyst preoperatively.

**Fig. 2:**
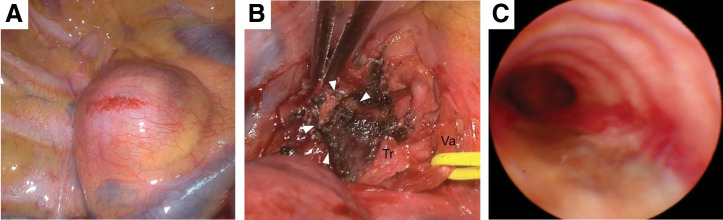
Intraoperative findings. (**A**) The tense cyst compresses the trachea and the esophagus. (**B**) Strong adhesion exists between the cyst and the membranous portion of the bronchus, with an indistinct boundary. A part of the cyst wall remains adherent to the trachea (arrowheads). (**C**) Bronchoscopic findings on POD 7 reveal white degeneration in the membranous portion of the trachea. Tr, trachea; Va, vagus nerve

Video-assisted thoracoscopic surgical resection of the mediastinal cyst was performed. Tracheal intubation with a double-lumen tube was challenging due to the tracheal stenosis. Additionally, the membranous trachea compressed by the cyst showed whitish degeneration on bronchoscopic findings, suggesting thinning and fragility. Tracheal intubation was performed carefully to avoid tracheal injury. The surgical approach was performed via an 8 cm lateral thoracotomy through the third intercostal space, with a port in the 7th intercostal space. Intraoperatively, the tense cyst was found to be compressing both the trachea and the esophagus (**Fig. 2A**). The cyst adhered firmly to both structures, particularly the membranous trachea, making complete resection difficult. Considering the thinning and fragility of the membranous trachea, we avoided aggressive dissection and deliberately left part of the cyst wall adherent to the trachea (**Fig. 2B**). Frozen section diagnosis was not performed because no solid component was observed in the cyst wall. To prevent the regrowth of the cyst due to mucus production from the residual cyst wall, the inner lining of the remaining cyst wall was cauterized using a VIO soft-coagulation system (Erbe Elektromedizin, Tübingen, Germany). Furthermore, to strengthen the fragile membranous trachea and to prevent recurrence by covering any residual mucosa, fibrin glue (Beriplast P Combi-set tissue adhesion, CSL Behring, Tokyo, Japan) and polyglycolic acid sheets (NEOVEIL Sheet, Gunze, Osaka, Japan) were placed over the trachea and the remaining cyst wall. Bronchoscopic findings on POD 7 showed improvement of the tracheal stenosis. However, the white degeneration of the membranous portion remained (**Fig. 2C**), requiring careful postoperative follow-up.

**Fig. 3:**
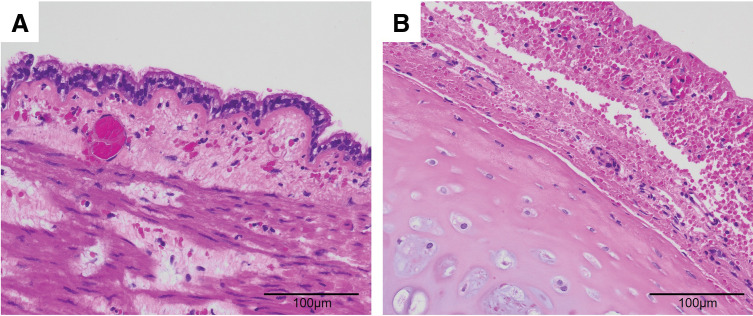
Histopathological findings of the mediastinal cyst. The hematoxylin-eosin stain (×200) demonstrates ciliated columnar epithelium on the luminal surface of the cyst (**A**) and cartilage on the cyst wall (**B**).

Histopathological examination confirmed the diagnosis of a bronchogenic cyst, revealing ciliated columnar epithelium lining the cyst cavity (**Fig. 3A**) and cartilage within the cyst wall (**Fig. 3B**).

At 6 months postoperatively, there was no evidence of cyst recurrence or tracheal perforation. The cough resolved, and the obstructive ventilatory impairment improved (FEV1.0:2.66 L, FEV1.0%:79.02%).

## DISCUSSION

In the present case, prolonged observation of a bronchogenic cyst until the onset of symptoms allowed it to enlarge and exert long-term compression on the membranous trachea, resulting in its thinning and weakening. Because the cyst was firmly adherent to the membranous trachea, forceful dissection was avoided to prevent injury to the thinned tracheal wall. This led to the inevitable decision to leave a part of the cyst wall in place. The present case highlights the importance of meticulously considering the surgery without delay and selecting appropriate surgical techniques.

Surgical resection of bronchogenic cysts should not be delayed due to the absence of symptoms. Many cases of asymptomatic mediastinal bronchogenic cysts are often observed until tumor enlargement or the onset of symptoms.^1)^ Patients may be reluctant to undergo surgery in the absence of symptoms. However, surgery for symptomatic bronchogenic cysts is associated with significantly higher rates of intraoperative complications compared with asymptomatic cases, including injury to adjacent organs.^3)^ In addition, the perforation of the cyst into the bronchus was reported.^1)^ Furthermore, if cyst infection occurs, it can form denser adhesions due to severe inflammation, making complete and safe surgical resection challenging.^4)^ These risks are more likely to arise with prolonged observation and are particularly concerning when the cyst is adjacent to vital organs, such as the left atrium, esophagus, or trachea. In our case, although the cyst enlargement over 10 years was not dramatic, it resulted in progressive tracheal stenosis, cough, and impaired respiratory function, which led to the decision to proceed with surgery. Furthermore, prolonged tracheal compression caused degeneration of the membranous trachea, likely contributing to its thinning and fragility. Tracheal stenosis complicated tracheal intubation, and the weakened membranous trachea increased the risk of iatrogenic injury during tracheal intubation and surgery. As a result, complete resection of the cyst was not achieved. Surgery should be performed before the cyst compresses adjacent structures or causes symptoms to achieve complete resection more safely and reliably. In retrospect, we should have performed surgical resection when the cyst was first identified 10 years ago.

Complete resection is recommended due to the risk of malignancy^5,6)^ and recurrence.^7–9)^ However, firm adhesion between cyst and adjacent organs can hinder complete resection. Histologically, these adhesions are associated with inflammation, hemorrhage, and fibrosis, suggesting the involvement of infarction and infection.^10)^ Moreover, the reported injury rate to adjacent organs is as high as 14%.^1)^ This high rate may reflect the fragility of neighboring tissues, as observed in our case. Therefore, it is important to preoperatively assess whether leaving a part of the cyst wall is acceptable, given the possibility of incomplete resection. In the present case, preoperative MRI and PET findings supported a low probability of coexisting malignancy. When a solid component is present, intraoperative frozen section diagnosis should be considered to rule out malignancy, although it was not performed in our case. Furthermore, bronchoscopic findings during tracheal intubation suggested fragility of the membranous trachea. Considering the high risk of tracheal injury with aggressive dissection, part of the cyst wall adherent to the trachea was intentionally preserved. This allowed for a safe procedure without injury to the adjacent organs. Nonetheless, the potential for late recurrence from the residual cyst wall remains a concern.^7–9)^ To reduce this risk, the inner surface of the preserved cyst wall was cauterized using an electrosurgical device.^2)^ A study in pediatric cases reported a low recurrence rate of 4.4% after partial cyst resection combined with cauterization or chemical obliteration of the residual mucosa using povidone-iodine.^11)^ Additionally, to reinforce the fragile membranous trachea and cover any residual mucosa that could lead to recurrence, we applied fibrin glue and a polyglycolic acid sheet over the trachea and the remaining cyst wall. These preventive procedures, though their efficacy has not been definitively established, may be considered in cases where complete resection is not achievable. Given the potential for late recurrence in cases of incomplete resection, long-term postoperative surveillance is required.

## CONCLUSIONS

To minimize the risk of intraoperative complications, surgical resection of bronchogenic cysts should ideally be performed before the patient becomes symptomatic. Complete resection can be difficult when there are firm adhesions to adjacent organs. Preoperative assessment for coexisting malignancy and abnormalities of surrounding structures can support surgical decision-making to ensure a safe procedure.

## DECLARATIONS

### Funding

Not applicable.

### Authors’ contributions

TH drafted the manuscript and collected clinical data.

MM and HI collected clinical data and revised the manuscript.

MK and YW were responsible for anesthetic management during the surgery and revised the manuscript.

AO diagnosed pathologically and revised the manuscript.

KF supervised this work and revised the manuscript.

All authors read and approved the final manuscript critically.

### Availability of data and materials

The datasets supporting the conclusions of this article are included within the article.

### Ethics approval and consent to participate

Not applicable. This work does not require ethical considerations or approval. Informed consent to participate in this study was obtained from the patient. It was conducted in accordance with the principles of the Declaration of Helsinki.

### Consent for publication

Written informed consent was obtained for the publication of this case report and accompanying images.

### Competing interests

The authors declare that they have no competing interests.
